# To infinity and beyond: the promise of data-driven 3D printing of hernia mesh – a primer for surgeons

**DOI:** 10.1007/s10029-025-03434-4

**Published:** 2025-09-01

**Authors:** Edward Young, James Lawson, Alex Karatassas, Chrys Hensman

**Affiliations:** 1 Department of Surgery, The Queen Elizabeth Hospital, The University of Adelaide, Adelaide, South Australia Australia; 2 The Basil Hetzel Institute, Woodville South, South Australia Australia; 3 School of Engineering, Ulster University, Belfast, Northern Ireland United Kingdom; 4 Swinburne University of Technology, University of Oceania, Melbourne, VIC Australia

**Keywords:** Hernia, Mesh, 3D printing, Integration, Index, Review

## Abstract

**Purpose:**

Abdominal wall hernias account for a substantial operative caseload in general surgery globally. Optimal hernia care should be tailored to individual circumstances. To repair the three-dimensional (3D) abdominal wall, 3D-printed patient-specific implants may be superior to current mesh products. The aim was to review the current state of 3D printing technology in custom hernia mesh production, and its safety and efficacy for tailored hernia care.

**Methods:**

A literature search within PubMed and Scopus databases were performed in March 2025, in accordance to PRISMA-ScR framework, using keyword combinations of printing, mesh, hernia, safety, efficacy and their derivatives. Full-text papers relevant to the study aim in all formats and languages were included, and risk of bias assessment was performed. The review was not eligible for registration with PROSPERO. Papers were grouped by general theme, and a narrative synthesis was performed.

**Results:**

Thirty relevant papers were identified from 14,210 abstracts. Literature on 3D-printed hernia mesh was sparse, with majority of papers being preclinical. General focus of the literature was production, cellular toxicity, performance of adjuncts and short-term tolerance in small animals. Risk of bias was globally high to critical, due to underreporting of in vitro and in vivo methodology. Safety and clinical efficacy of 3D-printed mesh remained unknown. Numerous issues, including production, sterilisation and regulations, were identified and discussed.

**Conclusion:**

3D-printed hernia mesh is the next step towards tailored hernia care, with significant potential not otherwise available with traditional mesh products. Substantial research is still required to clarify its safety and efficacy.

**Supplementary Information:**

The online version contains supplementary material available at 10.1007/s10029-025-03434-4.

## Introduction

Hernias are responsible for a substantial portion of operative caseload in general surgery [1]. Between 1990 and 2019, global hernia prevalence saw a 36% absolute rise, increasing from 23.9 million cases to 32.5 million cases [2]. Over a lifetime, one in four adult men are expected to develop an inguinal hernia [3, 4], and one in fifteen will develop an incisional hernia [5, 6]. Even with the best efforts of evidenced-based fascial closure techniques [7], incisional hernias continue to remain a material risk after abdominal wall surgeries [8, 9]. 

The current gold standard for hernia management is reduction of hernia sac, suture closure of defect and layer reinforcement with mesh [9]. Successful mesh repairs can be broadly understood as a two-step process, initiated by achieving mechanical compatibility in the short-term [10], and a subsequent transition to biomechanical stability in the long-term [11]. An unstable mesh tissue interface is prone to premature failure under physiological stresses and hinders cellular infiltration [12]. Mesh properties, such as effective porosity and immunogenicity, dictates the balance between tissue healing and foreign body fibrotic reaction [13–15]. Poorly shaped mesh, folding of mesh or mesh shrinkage negatively impact effective porosity, and is suspected to be a precipitator of chronic pain, seromas and hernia recurrence [11, 16]. 

Mounting evidence indicates that there is unlikely to be a ‘one size fits all’ option, and optimal hernia care should be tailored to individual circumstances [17]. The ideal hernia repair should be performed in a single planned operation with minimal disruption to local anatomical structures. Whilst the literature has generally focused on application of flat two-dimensional (2D) mesh, there is perhaps value in considering three-dimensional (3D) mesh as the next generation of repair materials. The abdominal wall is frequently approximated to a cylindrical structure out of convenience, yet it exhibits dynamic anisotropic behaviour during physiological activities, such as respiration, vomiting and coughing [12, 18]. It is unrealistic to expect that a uniformly woven 2D mesh will have 100% conformity to the surrounding dynamic 3D structures. Bending of 2D structures reduces effective porosity and places the repair at risk of poor mesh tissue integration. By conforming to local anatomy, 3D meshes have the potential to achieve better tissue integration and stability in the long term, and offers a promising avenue for patient-specific implant [19, 20]. 

Current 3D meshes approved for clinical use (e.g. Bard 3DMax™, Medtronic Parietex™ hydrophilic 3D mesh) are predominantly a multilayer construct, fabricated using traditional textile warp and weft knitting techniques and spacers. Marketed initially as fixation-free devices, the risk of migration, cost of implants and lack of long-term data have tempered initial enthusiasm [21–23]. This observation is perhaps less of a reflection of 3D mesh products, but more so on the fact that all hernia mesh repairs require some form of fixation or friction at the tissue interface, as demonstrated by Kallinowski’s critical resistance to impact pressure (CRIP) and gained resistance to impact pressure (GRIP) concepts [24]. Recent evidence suggests that short-term performance and behaviour of 3D meshes are on par with 2D meshes [25, 26]. While 3D contours can be produced using warp and weft knitting, the space-knitted domes continue to remain semi-generic and cannot precisely conform to patients’ individual anatomy. Since two abdominal walls are unlikely to have the same shape and resting physiological stresses, semi-generic knitted domes are not expected to achieve full conformity.

To achieve full conformity, a new method is required, such as 3D printing. 3D printing is an alternate pathway to creating anatomically contoured patient-specific mesh implants, and offers additional advantages not available with traditional textile techniques. The aim of this paper was to review the current state of 3D printing technology in custom hernia mesh production, and its safety and efficacy for tailored hernia care.

## Methodology

A literature search was performed in accordance to Preferred Reporting Items for Systematic Reviews and Meta-Analyses Extension for Scoping Reviews (PRISMA-ScR) framework. PubMed and Scopus databases were searched in March 2025 using keyword combinations of printing, mesh, hernia, safety, efficacy and their derivatives (Table 1). Citation list was also searched. Full-text papers relevant to the study aim in all formats and languages were included. All other papers were excluded (Table 2). Non-English papers were interpreted using Google Cloud Translate [27]. Risk of bias was assessed using the SYRCLE tool for animal studies [28], ROBINS-Intervention tool for non-randomised human studies [29], and the OHAT tool for in vitro studies [30]. This scoping review was not eligible for registration with PROSPERO. Papers were grouped by general theme, and a narrative synthesis was performed.

**Table 1:** Search strategy

PubMed	(printing[Title/Abstract]) AND (mesh*[Title/Abstract]) (printing[Title/Abstract]) AND (hernia*[Title/Abstract]) (printing[Title/Abstract]) AND (safety[Title/Abstract]) (printing[Title/Abstract]) AND (efficacy[Title/Abstract])
Scopus	(TITLE-ABS-KEY (printing) AND TITLE-ABS-KEY (mesh) (TITLE-ABS-KEY (printing) AND TITLE-ABS-KEY (hernia) (TITLE-ABS-KEY (printing) AND TITLE-ABS-KEY (safety) (TITLE-ABS-KEY (printing) AND TITLE-ABS-KEY (efficacy)
Citation Search	References of included articles

**Table 2:** Inclusion and exclusion criteria

Inclusion	Exclusion
Abdominal wall Hernia Mesh 3D printing/4D printing/additive manufacturing Safety Efficacy Any language Any date Any format	Full text not available Data not available Non-abdominal wall hernia mesh Does not discuss safety or efficacy

## Results

**Fig. 1:**
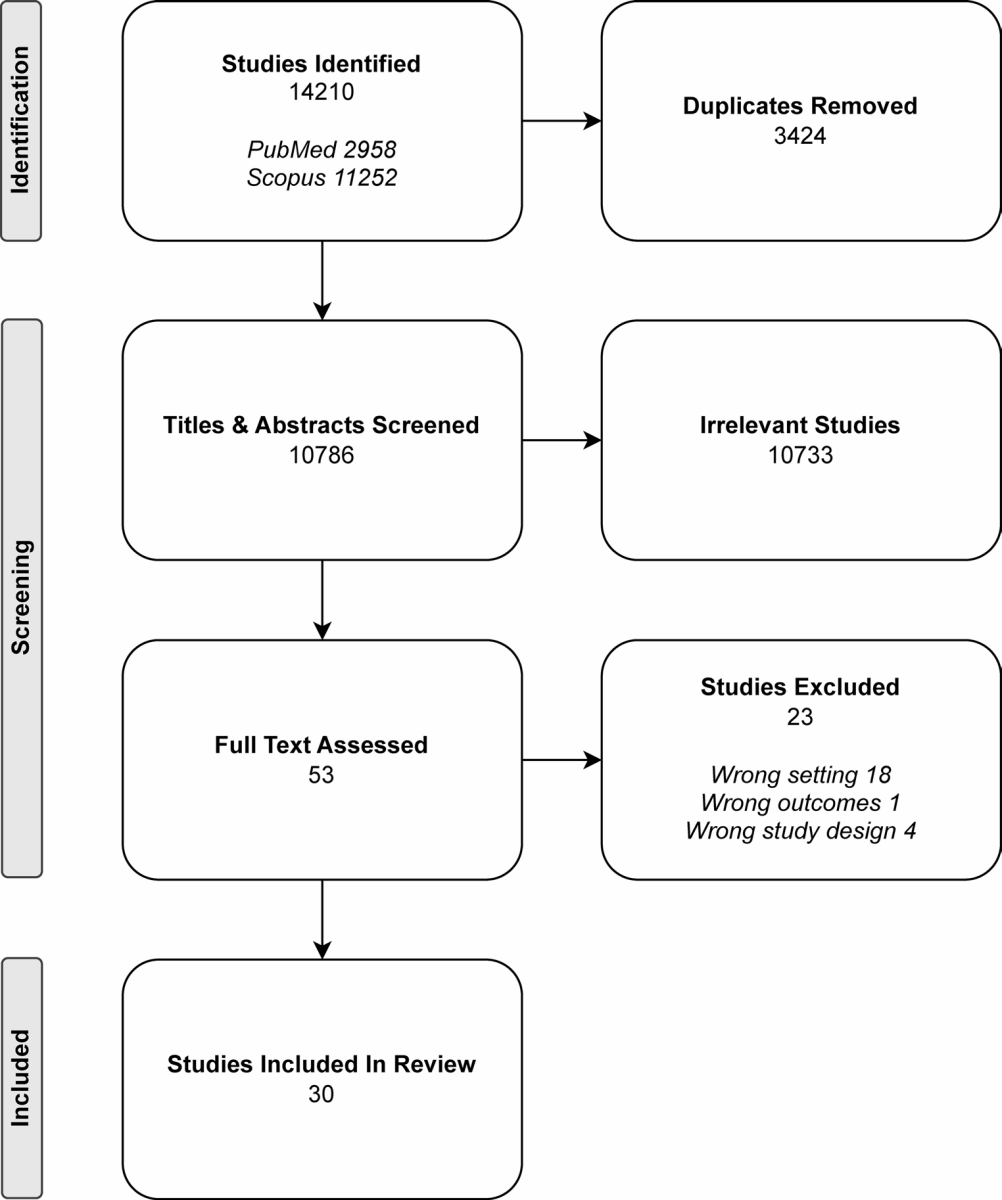
PRISMA chart

A total of 14,210 abstracts were identified from PubMed and Scopus. After excluding duplicates and irrelevant studies, 30 full-text papers were included (Fig. 1). No additional papers were identified from citation search. There were 23 experimental papers, 4 reviews, 2 book chapters and 2 perspectives (Tables 3, 4, 5 and 6). Government and university funding predominated, with minimal conflict of interest declared or evidence of biomedical industry involvement.

**Table 3:** Papers identified

Study ID	Title	Country	Funding level	Conflict of interest	Paper Type	Paper Focus
Ballard 2017	Three-dimensional printing of bioactive hernia meshes: In vitro proof of principle	United States	Not specified	None declared	Experimental	Mesh
Ballard 2018	3D printing of surgical hernia meshes impregnated with contrast agents: in vitro proof of concept with imaging characteristics on computed tomography	United States	Government	Declared	Experimental	Mesh
CaleroCastro 2019	Proof of concept, design, and manufacture via 3-D printing of a mesh with bactericidal capacity: Behaviour in vitro and in vivo	Spain	Not specified	None declared	Experimental	Mesh
Chen 2020	Tensile properties and corrosion resistance of PCL-based 3D printed composites	China	Government	Not stated	Experimental	Mesh
Corduas 2021	Next-generation surgical meshes for drug delivery and tissue engineering applications: materials, design and emerging manufacturing technologies	United Kingdom	University	None declared	Review	Mesh
Deveci 2024	Multifunctional hernia repair biopatch: Development, characterization, in vitro and in vivo evaluation	Turkey	University	None declared	Experimental	Mesh
Dykema 2019	Printing for the perfect fit: Balancing fda regulation of 3 d printed medical devices	United States	Not specified	Not specified	Perspective	Legal
Erwin 2023	Clinical observation, imaging, and histopathology of 3D polypropylene mesh for abdominal hernia in rabbits	Indonesia	University	Not specified	Experimental	Mesh
Feitshans 2022	3D PRINTED MEDICAL DEVICES: ISSUES FOR PATIENT SAFETY	United States	Not specified	Not specified	Perspective	Legal
Foster 2017	3-Dimensional Printing in Medicine: Hype, Hope, and the Challenge of Personalized Medicine	United States	Not specified	Not specified	Book Chapter	Legal
Galvan-Chacon 2021	3D Printed vs. Commercial Polypropylene Surgical Meshes: A Comparative Analysis of Tensile Strength	Spain	Not specified	Not specified	Experimental	Mesh
Garnica-Bohorquez 2023	Effect of Sterilization on the Dimensional and Mechanical Behavior of Polylactic Acid Pieces Produced by Fused Deposition Modeling	Colombia	Government	None declared	Experimental	Sterilisation
Georgantis 2019	Quality and safety in medical 3D printing	Greece	Not specified	Not specified	Book Chapter	Legal
Hu 2021	Topological Structure Design and Fabrication of Biocompatible PLA/TPU/ADM Mesh with Appropriate Elasticity for Hernia Repair	China	Government	None declared	Experimental	Mesh
Hu 2022	Designing Double-Layer Multimaterial Composite Patch Scaffold with Adhesion Resistance for Hernia Repair	China	Government	None declared	Experimental	Mesh
Hu 2024	3D printing/electrospinning of a bilayered composite patch with antibacterial and antiadhesive properties for repairing abdominal wall defects	China	Government	None declared	Experimental	Mesh
Olmos-Juste 2022	Tailor-Made 3D Printed Meshes of Alginate-Waterborne Polyurethane as Suitable Implants for Hernia Repair	Spain	Government	None declared	Experimental	Mesh
Perez-Kohler 2021	New insights into the application of 3d-printing technology in hernia repair	Spain	Government	None declared	Review	Mesh
Pettersson 2024	Core Legal Challenges for Medical 3D Printing in the EU	Finland	Government	None declared	Review	Legal
Qamar 2019	Personalized 3D printed ciprofloxacin impregnated meshes for the management of hernia	Pakistan	Not specified	None declared	Experimental	Mesh
Ramos 2023	Effectiveness in Sterilization of Objects Produced by 3D Printing with Polylactic Acid Material: Comparison Between Autoclave and Ethylene Oxide Methods	Brazil	None declared	None declared	Experimental	Sterilisation
RussoSerafini 2023	3D-Printed Medical-Grade Polycaprolactone (mPCL) Scaffold for the Surgical Treatment of Vaginal Prolapse and Abdominal Hernias	Australia	Government	Declared	Experimental	Mesh
Shea 2020	A review of the manufacturing process and infection rate of 3D-printed models and guides sterilized by hydrogen peroxide plasma and utilized intra-operatively	China	Private	None declared	Experimental	Sterilisation
Shin 2021	3D-Bioprinted Inflammation Modulating Polymer Scaffolds for Soft Tissue Repair	United States	Not specified	None declared	Experimental	Mesh
Smietanski 2023	Development and Implantation of 3D Anatomically Tailored Polypropylene Mesh for Laparoscopic Inguinal Hernia Repair Designed on the Basis of CT Images (the ILAM Study)	Poland	None declared	None declared	Experimental	Imaging
Song 2023	Reconstruction of Abdominal Wall Defect with Composite Scaffold of 3D Printed ADM/PLA in a Rat Model	China	Government	None declared	Experimental	Mesh
Sterk 2023	Development of New Surgical Mesh Geometries with Different Mechanical Properties Using the Design Freedom of 3D Printing	Portugal	Government	Not specified	Experimental	Mesh
Wang 2024	Polyurethane-based three-dimensional printing for biological mesh carriers	China	Government	None declared	Experimental	Mesh
Yadav 2025	Gelatin Multiwalled Carbon Nanotube Composite 3D Printed Semi Biological Mesh for Abdominal Hernia Treatment	India	Government	None declared	Experimental	Mesh
Yang 2020	A smart scaffold composed of three-dimensional printing and electrospinning techniques and its application in rat abdominal wall defects	China	Government	None declared	Experimental	Mesh

**Table 4:** Details of experimental papers examining 3D-printed hernia mesh

Study ID	3D Printing Method	Composition	Additives/Adjuncts	Mesh Size	Mesh Pore Size	Mesh Tensile Strength	Sterilisation	Ex vivo Testing	In vitro – Test Conditions	In vitro - Assessment	In vivo –Test Conditions	In vivo - Assessment
Ballard 2017	FDM	Polylactic acid	Gentamicin	Not specified	Not specified	Not specified	Not specified	No	Mueller-Hinton agar plates with E. Coli or S. Aureus, 37° Celsius, 24 h incubation	Zone of inhibition	No	-
Ballard 2018	FDM	Polycaprolactone	Barium Iodine Gadolinium	20 × 20 mm	Not specified	Not specified	Not specified	No	Sterile agar plate, 37° Celsius, 7 day incubation	Bacterial growth	No	-
CaleroCastro 2019	FDM	Polycaprolactone	Gentamicin Sodium alginate Calcium chloride	20 × 20 mm	1.25 × 1.25 mm 0.75 × 0.75 mm	Not specified	UV light/steam autoclave	No	Agar plates with E. Coli, 37° Celsius, 24 h incubation	Zone of inhibition	40 female Wistar rats, weight 236–281 g, with postmortem day 7	Histology, adhesion
Chen 2020	FDM	Polycaprolactone	Chitosan, hydroxyapatite Sodium alginate	Not specified	Not specified	Up to 18.7 MPa (1,870 N/cm^2^)	Potassium permanganate/sodium hypochlorite/acetic acid	Yes	No	-	No	-
Deveci 2024	FDM	Polycaprolactone	Ciprofloxacin Kappa carrageenan	15 × 15 mm	0.316 to 0.391 mm	Up to 3.73 MPa (373 N/cm^2^)	Not specified	Yes	Mueller-Hinton agar plates with a bacteria (S. Aureus, E. Coli, S. Epidermis or P. aeruginosa), 37° Celsius, 24 h incubation Human fibroblast cells (CCD-1072Sk), Dulbecco’s modified Eagle medium, incubated at 37° Celsius, with 5% CO2	Zone of inhibition Cell viability	56 male Wistar rats, 8–12 weeks old, weight 300–400 g, with postmortem day 14 and 28	Histology, adhesion, biochemical analysis
Erwin 2023	FDM	Polypropylene	None	100 × 100 mm	Not specified	Up to 321.67 kgf/mm^2^ (315,450 N/cm^2^)	Not specified	Yes	No	-	10 male New Zealand White Rabbits, 6–9 months old, weight 1–2 kg, with postmortem day 24, 48 and 96	Histology, ultrasound, biochemistry
Galvan-Chacon 2021	FDM	Polypropylene	None	Not specified	0.08–0.2 mm^2^	Up to 31.3 MPa (3,130 N/cm^2^)	Not specified	Yes	No	-	No	-
Hu 2021	FDM	Polylactide acid	Thermoplastic polyurethane/acellular dermal matrix		2.5 to 4 mm	Up to 17.3 N/cm, with 38.0% elongation	75% alcohol for 1 h	Yes	Human umbilical vein endothelial cells, in Dulbecco’s modified Eagle’s culture medium, incubated at 37° Celsius, with 5% CO2 for 1, 3 or 5 days.	Cell viability, cell proliferation	12 male Sprague-Dawley rats, weight 200 g, with postmortem day 28	Adhesion, histology
Hu 2022	FDM	Polycaprolactone	Polyvinyl alcohol + soy peptide	30 × 30 mm	Not specified	Up to 22.38 N/cm	Not specified	Yes	Human umbilical vein endothelial cells, in RPMI 1640 culture medium, incubated at 37° Celsius, with 5% CO2 for 3 days.	Cell viability, cell adhesion to mesh	12 male Sprague-Dawley rats, weight 180–200 g, with postmortem day 28	Adhesion, histology
Hu 2024	FDM	Polycaprolactone	Gelatine methacryl Sodium alginate Vancomycin	20 × 20 mm	Not specified	Up to 22.38 N/cm	Not specified	Yes	Human umbilical vein endothelial cells, in Dulbecco’s modified Eagle’s culture medium, incubated at 37° Celsius, with 5% CO2 for 1, 3 or 5 days.	Cell viability, cell proliferation, cell adhesion to mesh	12 male Sprague-Dawley rats, weight 180–200 g, with postmortem day 14	Adhesion, histology
Olmos-Juste 2022	FDM	Polyurethane	Chloramphenicol Sodium alginate Calcium chloride	50 × 58 mm	2.8 mm	Up to 27.60 N/cm, with 46.86% elongation	UV light for 30 min	Yes	L929 fibroblasts, in FBS culture medium, incubated at 37° Celsius, with 5% CO2 for 3 or 7 days	Cell viability	No	-
Qamar 2019	FDM	Polypropylene Polyvinyl alcohol	Ciprofloxacin	100 × 100 × 0.8 mm	< 3 mm	Up to 53 N/cm^2^ for polypropylene Up to 30 N/cm^2^ for polyvinyl alcohol	Not specified	Yes	No	-	20 male rabbit, weight 1 kg (species, postmortem time not specified)	Adhesion, histology
Russo Serafini 2023	FDM	Polycaprolactone	Platelet-rich plasma	30 × 30 mm for abdominal wall mesh	0.5 × 1 mm	Not specified	80% ethanol for 5 min, UV light for 20 min	No	No	-	6 sheep, postmortem month 3 and 6 (species, postmortem time not specified)	Biomechanical, histological, immunohistochemistry, scanning electron microscopy
Shin 2021	FDM	Polyvinyl alcohol	Sodium trimetaphosphate	Variable	Not specified	Up to 2.25 MPa (2,250 N/cm^2^)	Ethanol	Yes	Human dermal fibroblasts/human microvascular endothelial cells in Medium 106 and MCBD 131, incubated at 37° Celsius 5% CO2 for 72 h	Cell viability	6 female Balb/c mice, 10 weeks old, with postmortem at 5 days 6 Sprague-Dawley rats, 9–12 weeks, weight 300 g, with postmortem at weeks 2 and 4	Cytokine assessment, histology, adhesion
Song 2023	FDM	Polylactic acid	Acellular deceullarised matrix	25 × 25 mm	~ 0.5 mm	Up to 465.47 N/cm	Not specified	Yes	Human umbilical endothelial vein cell/rat skeletal muscle cell in L6 cell culture medium, incubated at 37° Celsius 5% CO2 for 48 h	CCK-8 cell proliferation	20 male Sprague-Dawley rats, weight 200 g, with postmortem at weeks 4 and 8	Histology, immunohistochemical staining, RNA expression
Sterk 2023	FDM	Polycaprolactone	None	Variable	Variable	Up to 16 N/cm	Not specified	Yes	No	-	No	-
Wang 2024	FDM	Polyurethane	None	Variable	Variable	Up to 32.7 MPa (3,270 N/cm^2^)	Not specified	Yes	Human cells HaCaT, HEK293T in RCTA culture medium, incubated at 37° Celsius, for 2–6 min	Cell viability	No	-
Yadav 2025	FDM	Gelatine	Penicillin/streptomycin	35 × 35 mm	1.0 mm	Up to 86 N/cm	70% ethanol for 1 h	Yes	L929 mouse fibroblast cell, in Dulbecco’s Modified Eagle medium, incubated at 37° Celsius, 5% CO2 for 3, 5 or 7 days	Cell viability, cell adhesion	No	-
Yang 2020	FDM	Polycaprolactone	None	Variable	0.36 to 0.48 mm	Up to 70 MPa (7,000 N/cm^2^)	75% alcohol for 1 h, sterilised by UV for 1 h	Yes	Rat dermal fibroblasts in Dulbecco’s modified Eagle’s medium, incubated at 37° Celsius, 5% CO2 for 1, 3 or 5 days	Cell viability, cell proliferation	60 Sprague-Dawley rats, weight 200–250 g, postmortem weeks 2 and 4	Histology, biomechanical

*FDM* fused deposition modelling

E. Coli: Escherichia coli

*S. Aureus: Staphylococcus aureus*

*S. Epidermis: Staphylococcus epidermis*

*P. aeruginosa: Pseudomonas aeruginosa*

**Table 5:** Details of experimental papers examining sterilisation of 3D-printed hernia mesh

Study ID	3D Printing Method	Composition	Additives/Adjuncts	Mesh Size	Mesh Pore Size	Sterilisation	Ex vivo Testing	In vitro – Test Conditions	In vitro - Assessment	In vivo –Test Conditions	In vivo - Assessment
Garnica-Bohorquez 2023	FDM	Polylactic acid	None	115 × 19 mm	Variable	Formaldehyde with steam autoclave	Yes	No	-	No	-
Ramos 2023	FDM	Polylactic acid	None	Variable	Not specified	Steam autoclave/ethylene oxide	No	Brain heart infusion broth, at 34–37° Celsius, incubate for 48 h or 15 days. Then MacConkey agar plate at 34–37° Celsius for 24 h.	Bacterial Growth	No	-
Shea 2020	FDM	ABS-M30i	None	Variable	Not specified	Vaporised hydrogen peroxide gas plasma	No	No	-	121 adult humans, implantation of 3D-printed items, with clinical follow up > 3 months	Clinical follow up, complication rates

*FDM* fused deposition modelling

**Table 6:** Details of experimental papers examining medical imaging and 3D-printed hernia mesh

Study ID	3D Printing Method	Composition	Additives/Adjuncts	Mesh Size	Mesh Pore Size	Sterilisation	Ex vivo Testing	In vitro – Test Conditions	In vitro - Assessment	In vivo –Test Conditions	In vivo - Assessment
Smietanski 2023	-	Polypropylene	*existing mesh shaped over 3D-printed model	Variable	Variable	Not specified	Yes	No	-	3 adult humans, with implantation of 3D-printed items, with follow-up at 7 days, 3 months and 12 months	Clinical follow up, complication rates

**Fig. 2:**
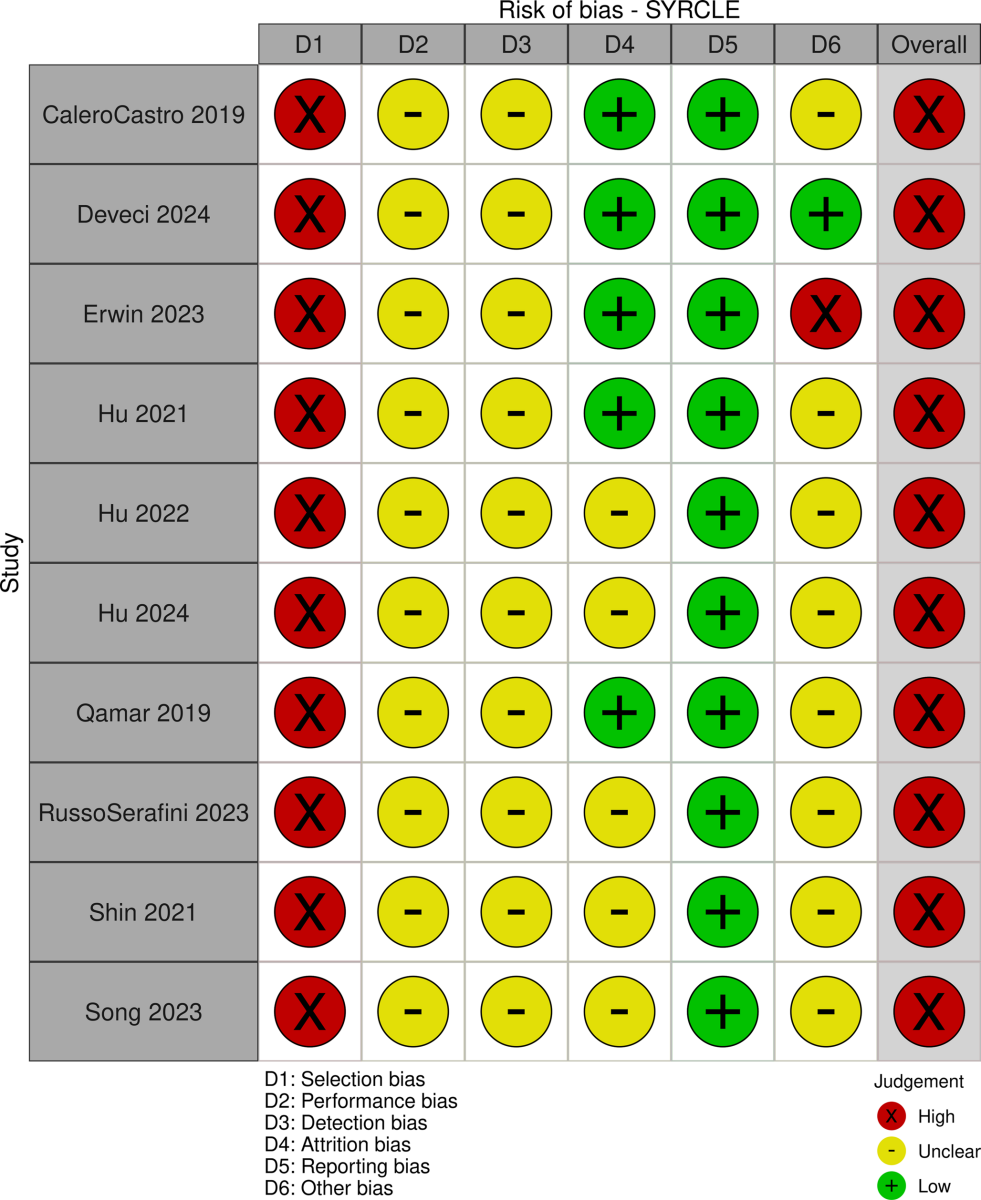
Risk of bias in in vivo animal experimental papers using Hooijmans’ SYRCLE tool. [28] Image produced using McGuinne’s RobVis Tool. [31]

**Fig. 3:**
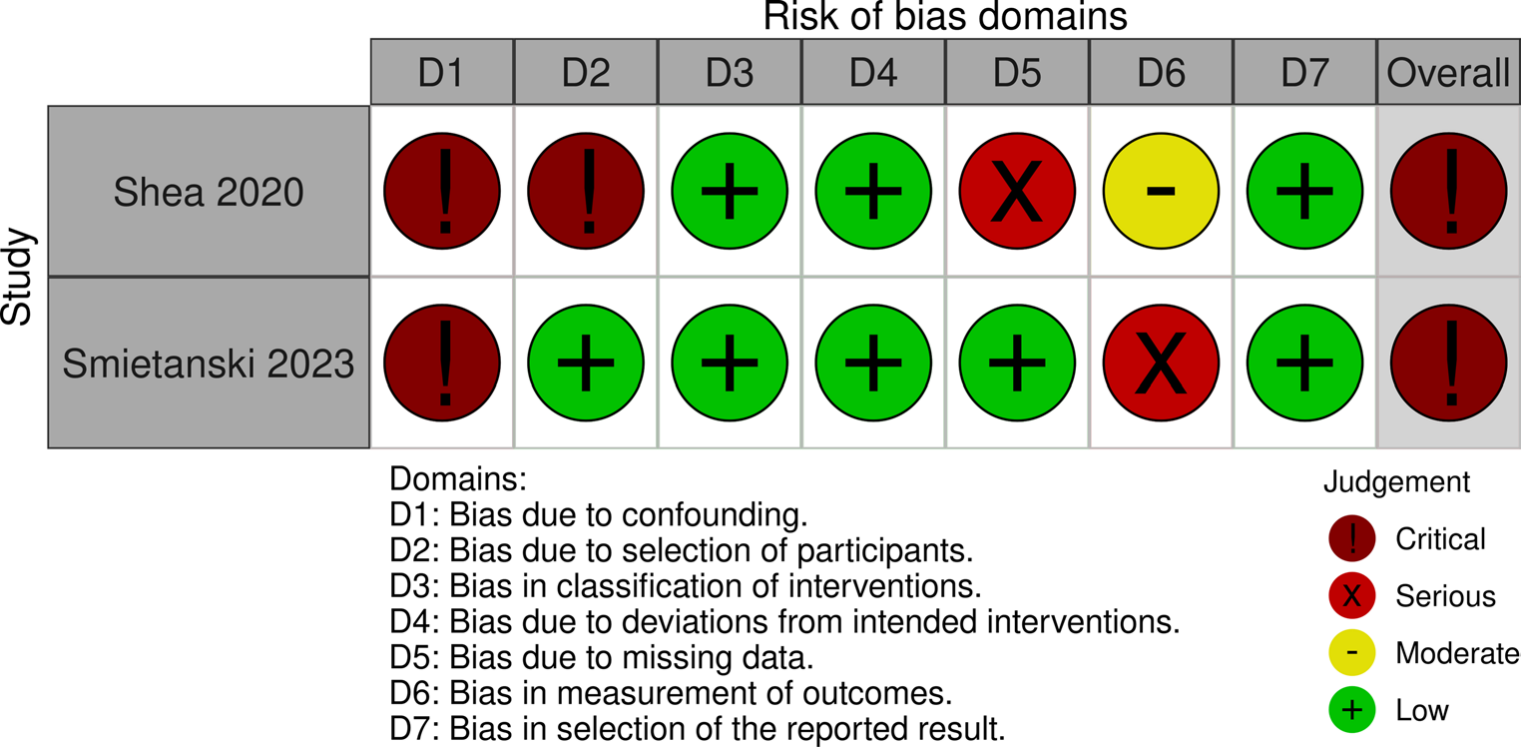
Risk of bias in in vivo human experimental papers using ROBIN-Intervention tool. [29] Image produced using McGuinne’s RobVis Tool. [31]

**Fig. 4:**
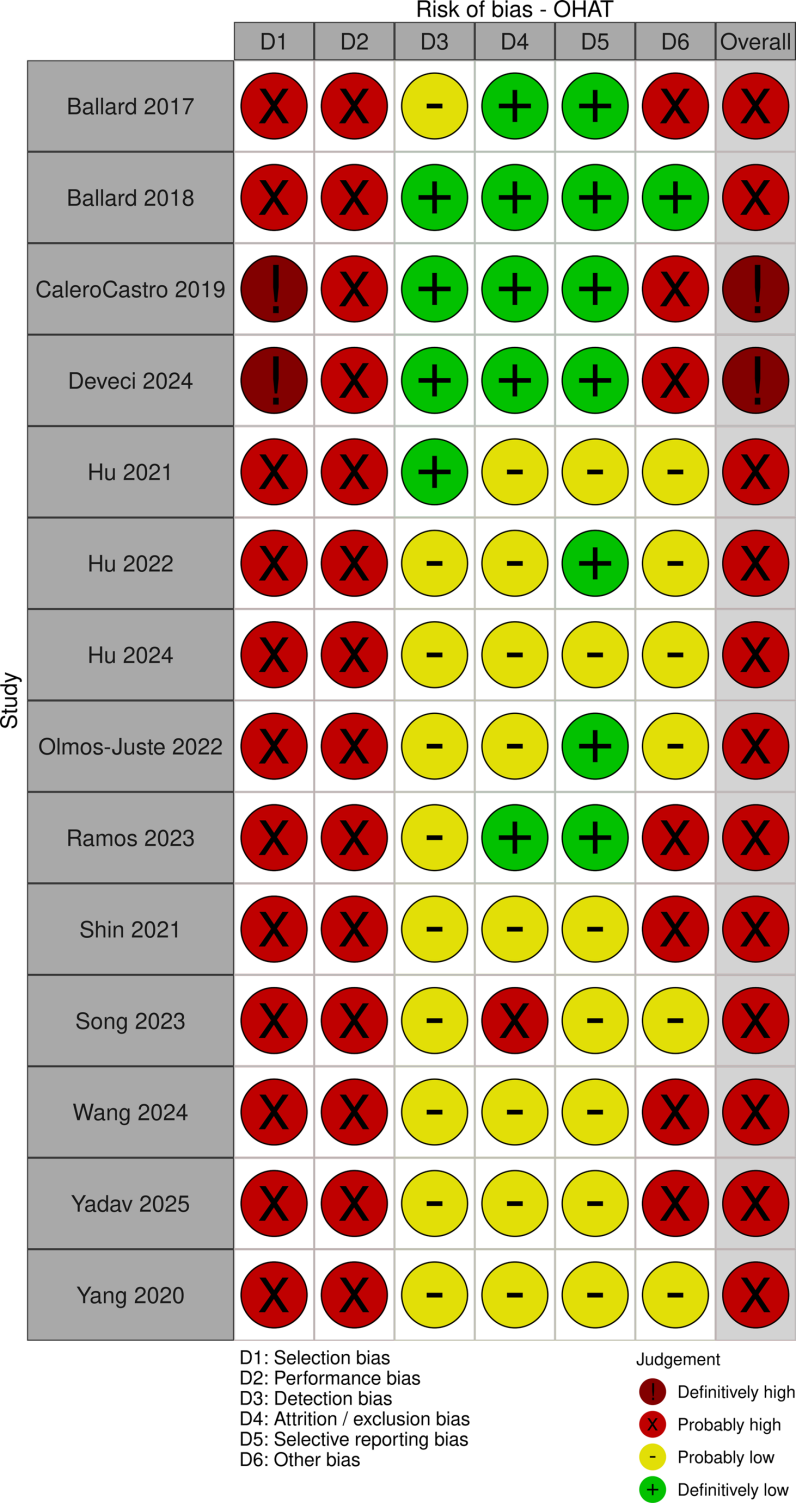
Risk of bias in in vitro experimental papers using National Toxicology Program OHAT tool. [30] Image produced using McGuinne’s RobVis Tool. [31]

All experimental papers, both in vivo and in vitro, scored poorly with risk of bias assessment, with high to critical risk (Figs. 2, 3 and 4). Some papers required multiple risk of bias tools for assessment, due to inclusion of both in vitro and in vivo study components (Supplementary 1). Although all papers provided excellent technical information on 3D printing and engineering testing protocols, the overall reporting quality of in vitro and in vivo experiments were poor and highly concerning.

The ARRIVE guidelines were largely not followed for in vivo animal studies, with the omission of essential information [32]. All experimental studies failed to report whether samples or specimens were randomised and how randomisation was performed (randomisation table or computerised randomisation). No study reported whether assessors were blinded, whether animals or specimens were concealed, and whether animals were housed randomly. Authors often did not clearly state whether assessments were performed in same fashion for all animals or specimens, whether there were any deaths or drops outs during the experimental phase, and whether there were any difficulties with the assessment process. No study clearly stated who performed histology assessments, and whether they were blinded from the study to maintain objectivity.

This observation was largely the same for in vitro studies. While appropriate controls were reported in most studies, there was no clear description of attempts to reduce bias from investigators, such as steps of randomisation, blinding assessors when performing cell viability assessments, or when measuring zones of inhibition for antibiotic susceptibility tests. Raw data is often not included in the manuscript or as supplementary materials, and requires readers to contact the authors.

Substantial text in all experimental studies were devoted to discussing results, statistical significance and general interpretation of results. No paper seriously reflected upon potential risk of bias, error or design flaw within their studies.

## Discussion

The literature on 3D-printed hernia mesh is relatively new and expanding. In 2021, Corduas et al. and Perez-Kohler et al. independently reviewed the state of hernia mesh and the role of 3D printing technology in medicine [33, 34]. Since then, 13 additional experimental papers specific to 3D-printed hernia mesh have been published by a variety of author groups across the world. (Tables 3 and 4)

While these are promising advancements, greater emphasis needs to be placed upon study design and transparent reporting of results, by following the ARRIVE guidelines or equivalent for in vitro and in vivo components. Many papers often end up combining both in vitro and in vivo methodology, without clear explanation as to why both components were performed. If the design and experiments were performed in a stepwise fashion, i.e. in vitro before in vivo, then this should be clearly explained and documented. Likewise, if the purpose of in vitro study was for toxicology, then the appropriate toxicology reporting standards should be followed to minimise risk of bias, such as following the guidelines set out by the European Union Reference Laboratory for Alternatives to Animal Testing (EURL ECVAM) and the TOXR tool [35, 36]. As evident in the risk of bias assessments conducted, information necessary to guarantee that the results were bias-free were largely absent. There was a substantial barrier to interpretation of study results, as high to critical risk of bias generally is not usable.

What can be safely interpreted from the identified experimental papers, is that there are many ways to print 3D meshes, adjuncts may be added to the mesh during production, and such mesh may elicit a desirable in vitro or in vivo response. The ability to incorporate antibiotic function has potential and could be an answer to minimising mesh infection and biofilm development. In non-experimental papers, authors are increasingly voicing concerns regarding regulating 3D-printed medical devices produced at point of care.

### What is 3D printing?

3D printing is a form of additive manufacturing that has been in existence for some time. Raw materials suitable for 3D printing include metals, ceramics, paper and polymers. Using computer-aided design (CAD) software, a desirable object is digitally created and saved as a CAD file. CAD files may also be created by scanning objects using specialised laser equipment, or by reconstructing objects from radiological imaging, such as Digital Imaging and Communications in Medicine (DICOM) files. Using CAD files, segmentation software then digitally slices the object into thin layers and creates a printable instruction file known as G-code. G-code is then transmitted to 3D printers, and the object is created [37]. 

Many types of 3D printing are available on the market, with the difference primarily being how the raw material is prepared and how it is bound together to form the object. Some 3D printers, such as electron beam melting (EBM) or direct energy deposition (DED) are only suitable for the creation of metallic or ceramic objects, typically used in automobile industry or aerospace engineering. The following 3D printers are some examples with relevance to producing custom hernia mesh.

Fused filament fabrication (FFF), also known as Fused Deposition Modelling (FDM), is one of the initial 3D printers developed and was first patented in 1989 [38]. FDM has experienced rapid growth in the technology in recent decades [39], primarily due to use in rapid prototyping [40]. Rapid prototyping is the designing and printing models of an object or feature within a short time frame, often as a sample (prototype) to a bigger project. In FDM, once G-code has been transmitted to the 3D printer, polymer filaments are heated to a liquid state, extruded through a nozzle and deposited layer by layer. Specific heating and nozzle settings for a given polymer material are supplied by filament manufacturers [41]. FDM is considered user-friendly and has a low setup cost. It can use a range of common thermoplastics at relatively low temperatures, such as polylactic acid (PLA), acrylonitrile butadiene styrene (ABS), polyethylene terephthalate glycol (PETG) or thermoplastic polyurethane (TPU) [42]. High-end FDM printers can achieve greater temperatures, allowing for the use of high-performance polymers such as polyether ether ketone (PEEK), polyetherimide (PEI) or carbon fibre composites [43]. FDM is versatile, and a good introduction to 3D printing for novices. Drawbacks of FDM include loss of dimensional accuracy during printing, printing may be time consuming for complex geometry, and post-processing (e.g. sandpapering or milling) may be required to achieve the desired surface finish.

Stereolithography (SLA) uses an ultraviolet (UV) laser to cure liquid resin layer by layer, allowing finer detail and smoother prints to be achieved [44]. Objects generally require post-processing, including curing in a UV chamber to further harden the object [45], and application of isopropyl alcohol (IPA) to remove surface tackiness [46]. 3D-printed resin models are commonly used for teaching purposes, to demonstrate patient-specific anatomy or for pre-operative planning. Most resins are epoxy-based with carcinogenic properties, and thus are considered too toxic for implant applications [47, 48]. 

Selective laser sintering (SLS) uses lasers to fuse powder particles layer by layer. Powder can be metallic, nylon or TPU. Without the need for support structures, complex geometries and intricate internal structures may be created [49, 50]. SLS is well suited to produce hernia mesh and surgical-grade implants. SLS is currently only available in industrial-size platforms, and initial set-up costs may be considered prohibitive [51]. 

Bioprinting is a fusion of 3D printing technology and tissue engineering [52]. Instead of using inorganic materials, bioprinting uses bioink. Bioink is a water-based hydrogel that mimics the extracellular matrix, and typically incorporates cells and biochemicals, such as growth factors and cytokines, to support vascularisation and tissue growth [53–56]. Bioprinting have been used experimentally to produce custom organs, and has immense potential in regenerative and transplant medicine [57]. Bioprinting could theoretically be used to create custom biological hernia meshes with bioactive properties that elicits desirable immunological responses to initiate healing, with minimal foreign body response.

Commonly used medical-grade polymers include polycaprolactone (PLC) [58–66], polylactic acid (PLA) [67–69], polyvinyl alcohol (PVA) [70, 71], polypropylene (PP) [70, 72], polyurethane (PU) [2, 73] and gelatine carbon nanotubes [74]. These polymers have minimal toxicity, are well established in the medical industry, and can be easily converted into printable 3D filament with a filament extruder.

### 3D-printed patient-specific implants

3D printing has created an alternate pathway towards creation of affordable custom biomedical devices, a feat previously not possible with traditional manufacturing techniques. The high degree of customisation offered by CAD allows designing and manufacturing processes to be driven predominantly by clinician expertise and patient circumstances, creating a collaborative environment to produce patient-specific implants and tailored healthcare. Patient-specific implants for orthopaedic, dental and facial reconstruction have been shown to achieve faster functional return and greater patient satisfaction [75–78]. Detailed anatomical models have been invaluable to surgical education and simulations [79–81]. 

In the case of abdominal wall hernia management, a range of customisable features may be possible [59–74, 82, 83]. The size, contour and shape of the mesh could be predetermined based on preoperative imaging, using either computed tomography (CT) or magnetic resonance imaging (MRI) [84]. This could remove the need to trim meshes intraoperatively, decrease handling time and reduce risk of bacterial contamination. Optimal filament size and mesh thickness may be predetermined based on expected wall stresses and desired effective porosity. The thickness of the mesh may be varied such that it has adequate strength over maximal stress regions, as dictated by fracture mechanics, while minimising mesh burden [18]. Specific mesh material may be chosen based on expected activity levels [84]. For example a flexible bioabsorbable polymer may be better suited for a hernia repair in a young patient who is expected to lead a more active lifestyle.

Mesh surface topography can be laser etched to dissuade bacterial colony attachment and subsequent biofilm formation [85]. Use of plasma processing could increase surface hydrophilic properties to encourage cell attachment [86, 87]. Radiopaque contrast may be incorporated into mesh fibres to allow better visualisation on subsequent medical imaging for follow-up or diagnostic purposes [59]. Likewise, drug release microcapsules, bacteriophages, zinc, copper or silver ions could be incorporated into the mesh structure to provide antibacterial properties, and potentially reduce post-operative infections [62, 64, 67, 70, 73, 74, 88, 89]. A new generation of ‘intelligent’ meshes may be possible by imbedding special sensors in the mesh structure to detect infection or monitor healing [90]. 

The potential of 3D-printed meshes may also be extended to a broader range of indications where meshes have been conventionally used. Pelvic floor reconstructions are notoriously difficult to perform due to geometric shape, dynamic structures, and prominent neurovascular bundles [91]. Patient-specific 3D-printed implants could reduce the difficult of such operations, and facilitate local tissue integration with adjuncts built into the mesh structure. Similarly, congenital hernias in paediatric patients require accommodation of body growth when planning surgical repairs, and 3D-printed implants could offer improved outcomes [92]. 

The utility of 3D-printed patient-specific implants is likely applicable to all surgical disciplines that require individualised anatomically responsive solutions. With sufficient trained personnel and equipment, patient-specific implants could be produced onsite within a healthcare facility [19, 93]. 3D printing is an attractive consideration by centres in developing regions where normal procurement may be difficult due to logistic supply chain issues [94]. 

### Current state of 3D-printed hernia mesh

Clinical use of 3D-printed hernia mesh is currently limited to case-by-case provision [84]. The vast majority of published papers are preclinical and focus on describing mesh production techniques, in vitro toxicity, performance of adjuncts (e.g. antimicrobial properties) and short-term tolerance by small mammals [60, 62–64, 68–71, 82]. Only one study used a large animal model, namely sheep [65]. While there is nothing inherently wrong with using small animal models, and perhaps encouraged from an animal welfare point of view when investigating new substances and products [95], in vivo outcomes observed in small animals typically do not translate well to performance in large animals or humans [96]. Large animal studies over medium- to long-term are necessary to characterise in vivo behaviour of 3D-printed mesh products, as mandated by the International Organization for Standardization 10993-6 [97]. 

The meshes printed so far are relatively small. Of the studies that reported mesh size, only two studies implanted meshes larger than 5 × 5 cm [70, 82]. Uniaxial tensile testing of printed mesh was reported in 15 out of 19 experimental studies. Of which, only 3 studies compared their tensile strength to the estimated longitudinal stress of 16 N/cm and hoop stress of 32 N/cm in the abdominal wall, as derived by Klinge et al. [98] Works by Kallinowski’s group indicate intra-abdominal pressures during intense post-operative vomiting may be as high as 225 mmHg [99]. This rapid change in pressure could theoretically generate a hoop stress of 48 N/cm^2^ along the abdominal wall, or about 48 N/cm for every 1 cm length of tissue [100]. The 3D-printed meshes of some studies are likely to fracture before they can obtain the necessary GRIP values to overcome the CRIP values of defects [24]. Coined by Kallinowski, GRIP is a numerical representation of the stability of mesh fixation and surface friction it has to resist against cyclical impacts, while CRIP describes the minimum level of GRIP required to resist against a standardised quantity of cyclical impacts that simulates intra-abdominal pressures generated by post-operative coughing. On the other hand, some meshes are likely overengineered, such as Erwin et al.’s 3D-printed polypropylene mesh that has a reported tensile strength of 321.67 kgf/mm^2^ (~ 315,000 N/cm^2^) which is on par with Kevlar fibres (362,000 N/cm^2^) used in ballistic body armour [101]. 

Elasticity is another consideration and appears to be poorly documented. Only two studies clearly reported elongation of 3D-printed mesh under strain [68, 73]. The abdominal wall has an inherent degree of elasticity (about 32% at 16 N/cm) which the mesh should ideally match [102]. This is influenced by the construct of the mesh, with lightweight mesh (35–70 g/m^2^) stretching more than heavyweight mesh (≥ 140 g/m^2^) [103, 104]. Meshes with elongation rates greater than the native abdominal wall, i.e. >30%, may not maintain functional repair and could be a cause for repair failure in the long-term [100]. Excess elasticity can also alter effective porosity [105]. While several studies reported textile porosity over 1 mm^2^ [60, 62, 68, 70, 73, 74, 106], which is the minimum pore size needed to prevent the bridging effect from fibrotic tissue [107], no study specified whether these values were under static or dynamic conditions. Loss of effective porosity prevents ingrowth of tissue and leads to excessive scar plate formation, which may precipitate development of seroma, chronic pain and repair failure [11, 13]. 

Another problem is long-term stability of mesh. The most commonly reported materials used to fabricate 3D-printed meshes were PCL and PLA (Table 4). Both of these substances have well-established biological safety and immune profiles [33]. PCL is a copolymer of the monofilament suture Monocryl^®^ (polycaprolactone/polyglycolide) (Ethicon Inc, New Jersey, United States), while PLA is the primary component of the ‘Velcro-like’ grips in ProGrip^®^ mesh (Medtronic Australia Pty Ltd, New South Wales, Australia). Both PCL and PLA are classified as biodegradable polymers. These substances are currently only used as adjuncts or as a copolymer in regulatory-approved hernia mesh, and not in their pure forms. Although PCL is reported to have a ‘slow degradation’ over 2–4 years [108, 109], concerns have been raised that electrospun fibres have accelerated degradation when exposed to hydrolytic enzymatic action, and tensile strength loss may be encountered as early as 90 days [110]. The manufacturer data on Monocryl^®^ indicates an expected loss of strength over 14 days, and complete resorption by 90–120 days. Investigations into using PLA as a suture material found 12% reduction in knot strength after submersion in normal saline at room temperature for 28 days [111]. A polymer mixture or composite structure may be required to meet the biomechanical demands of hernia repairs, while still having low immunogenicity and biodegradative properties.

Since hernia meshes are long-term implantable devices, sterility of the mesh must be guaranteed. It is suspected that bacterial contamination, such as by Staphylococcus aureus [112], and subsequent biofilm formation from sub-acute infection is responsible for a portion of repair failures years after surgery [113]. In most of the experimental papers, if described, have only used submersion in concentrated ethanol and UV light exposure as a sterilisation method (Tables 4 and 5). These methods should be more appropriately termed disinfection and are inadequate for sterilising critical devices, such as surgical implants, that enter sterile regions of the body, due to their inability to kill and remove bacterial spores [114, 115]. A sterilisation study of 3D-printed objects noted that hollowed objects could not be fully sterilised with either steam autoclave or ethylene oxide (EO), with non-coagulase Staphylococcus species continued to be isolated post-sterilisation [116]. Likewise, Garnica-Bohorquez et al. did not find any success in a two-step formaldehyde steam autoclave and noted significant loss in tensile strength of 3D-printed PLA meshes. Shea et al. tested low-temperature vaporised hydrogen peroxide (VHP) gas plasma sterilisation for a variety of 3D-printed objects for clinical use, and recorded a surgical site infection rate of 7.0% (8 of 114 patients), with 5 patients (62.5%) requiring surgical debridement or implant removal/revision [117]. Although the temperature of printing processes typically exceeds 200° Celsius, a substantial amount of post-printing processing is expected and opens up multiple routes and opportunities for contamination [117]. The surface topography of 3D-printed objects is irregular, and like textured breast implants or healthcare surfaces, provides the ideal environment for bacterial attachment and biofilm formation [118, 119]. 

EO and VHP are standard methods of sterilisation in the central sterilisation services department (CSSD) in hospitals, with typical operating temperatures of 50–60° Celsius. This is close or substantial above the glass-transition temperatures of PLA or PCL, which are 60–65° Celsius and minus 60° Celsius, respectively [120]. Sterilising with EO or VHP using the current protocols will likely lead to deformation and surface damage of 3D-printed objects [120]. Currently, it appears STERIS Healthcare (Dublin, Ireland) is the only company providing a dedicated sterilisation cabinet for 3D-printed objects using VHP (V-Pro Max 2 system), in conjunction with proprietary resin materials. It is unclear whether such resin materials are suitable for hernia mesh production. Choice of mesh printing material needs to take into consideration of method of sterilisation, and the potential physiochemical reactions that may occur.

### Safety and efficacy of 3D-printed hernia mesh

Without large animal model and clinical studies, it is difficult to state whether 3D-printed mesh will be efficacious to the management of abdominal wall hernias. The experiments identified so far indicates that there is no immediate toxicity or handling concerns. Histological analysis consistently shows expected acute phase inflammatory reaction [60, 62–65, 68, 70, 71, 82, 106]. No further meaningful conclusions could be drawn due to high risk of bias from unclear randomisation process of animals involved (Fig. 2). To maximise data reliability, the PREPARE and ARRIVE guidelines should be followed when designing and reporting animal studies, and the equivalent guidelines for in vitro studies [32, 121]. Pre-registration of animal protocols is strongly recommended to ensure study integrity and minimisation of biases.

A critical element in evaluating whether a hernia mesh function as intended, is to assess the interface between the mesh and tissue, and to examine how well tissue integrates or infiltrates over time. Good tissue integration is a reflection of beneficial tissue growth and minimal foreign body response, and corresponds with strong biomechanical stability that is necessary at preventing long-term hernia recurrence. To assess mesh tissue integration in an objective fashion, a standardised Mesh Integration Index was previously proposed and validated in a large animal model [manuscript under consideration] [122]. The Index allows preclinical standardised assessment of mesh performance, providing the necessary information to inform researchers whether a mesh function as intended. Problematic meshes could be identified and recalled prior to marketing, and thereby minimising harm to patients. The Index is applicable to all mesh products in the abdominal wall, including 3D-printed meshes.

Briefly, the Index uses a series of standardised assessments to grade integration, fibrosis, degradation and adhesion on a 0 to 5 ratio scale. The assessments incorporate visual, histological, biomechanical and molecular tools. These tools were selected due to their widely available in standard biomedical research institutes or laboratories, which allows rapid assessment of in vivo results using standard animal models. The in-vivo behaviour of 3D-printed meshes is easily quantified by the Index, streamlining comparison of different 3D-printer compounds and discovery of new mixtures or methods to produce clinically impactful hernia meshes. The secondary purpose of the Index is to provide a uniform language between biomaterial scientists, who are likely involved in the designing of the product, and clinicians, who are ultimately the end users of mesh products.

The ideal mesh is one that achieves high levels of tissue integration in the shortest amount of time after implantation and has the least amount of foreign body fibrotic reaction. The mesh should conform to the structure of the local anatomical structures, have sufficient elasticity, maximal effective porosity, antibacterial properties and sufficient strength to accommodate the dynamic physiological stresses in the abdominal wall. Meshes that intend to be intraperitoneal should have anti-adhesion properties that do not interfere with tissue integration. Meshes should ideally have minimal degradation with no loss of tensile strength for at least 5 years from time of implantation. Meshes should preferably be relatively easy to handle, can tolerate intraoperative manipulations, conform to local anatomy and not require trimming.

Like any other medical device, 3D-printed mesh, if deployed to clinical use, should be tracked and monitored with a clinical quality registry that incorporates a device registry. Outcomes of lesser-known products should be monitored, such that adverse events can be detected at the first instance, to minimise harm to patients. Registries and big data platforms are vital to tracking real‑world effectiveness and complications (e.g. China’s national hernia registry with ~ 100,000 cases) [123]. Ultimately, device regulations should occur at a national level, through regulatory authorities, such as the United States Food and Drug Administration (FDA), the European Union (EU), or the Australian Therapeutic Goods and Administration (TGA).

### Regulations and ethics of 3D-printed hernia mesh

Medical device regulation has traditionally been manufacturer heavy, with the onus on only approving devices that meet the necessary standards for safety and efficacy as set by the local regulatory authority. Devices undergo a series of standardised testing to ensure that that it is not toxic to humans, it achieves its intended purpose, and it does not have long-term side effects or potential problems that may arise from device malfunction. Devices need to be manufactured to a minimum standard that meets national and international regulations, ensuring integrity and consistency of the device, be adequately sterile for its purposes, and have quality assurance processes in place to detect problems. 3D-printed hernia meshes currently falls into a grey area within legislation, as it can be both mass-produced in terms of numbers and has a degree of customisation to fit the patient for usage [124, 125].

In the US, oversight relies on the FDA’s Custom Device Exemption (CDE), such as Investigational Device Exemption (IDE) via Centre for Devices and Radiological Health (CDRH) or the 510(k) Premarket Approval (PMA) pathway. The latter is optimised for conventional ‘substantially equivalent’ devices. Under the CDE pathway, a 3D-printed device may qualify as exempt from standard premarket approval if it meets specific criteria: it must be custom-made in direct response to a written request from an authorised health professional (e.g. a surgeon); the design must originate from the clinician and not be substantially manufactured in advance by the device producer; often, design input may include patient-specific templates, scans, or clinical sketches provided by the health professional [126]. Truly personalised meshes may struggle to fit existing categories but if the mesh is fully unique per patient (e.g. from a CT scan-derived 3D model) and fabricated as a once off, CDE may apply but this cannot be used for scaling up or commercialisation under the 21 Code of Federal Regulation (CFR) 812.3(b). If a 3D-printed mesh has no predicate but poses low to moderate risk, de novo classification may be achieved instead via US Federal Food Drug and Cosmetics Act (FDCA) Sect. 513(f)(2) [127]. Dykema argues that a new scheme should be created by the FDA under the existing Class III medical devices regulatory classification that is specific to custom 3D-printed devices and contains elements designed specifically to address the unique nature of additive manufacturing at point of care [124]. 

In the EU, 3D‑printed devices fall under the Medical Device Regulation (MDR). The EU MDR (2017/745/European Union) outlines the framework for regulation, mandating a bare minimum of clinical evaluation, risk management, quality management system, post-market surveillance, technical documentation, and liability for defective devices [128]. A submission for use to MDR requires detailed technical documentation and clinical justification equivalent to major implants. This places significant demands on healthcare facilities to coordinate multidisciplinary in‑house teams, certified under International Organization of Standardization (ISO) 13,485, if 3D printing was to be performed at point of care [129]. Pettersson critiques that the MDR does not truly address hospitals as manufacturers, and there continue to lie problems of ownership and usage of intellectual properties of devices and software, not withstanding liability issues [129]. 

In Australia, surgical meshes were reclassified by the TGA from Class IIb (medium risk) to Class III (high risk) devices in 2018, and fully implemented by 2021 [130]. These changes mandated a full TGA conformity assessment and inclusion within the Australian Register of Therapeutic Goods (ARTG), removing the previous lenient pathway for implant approval [131, 132]. The TGA currently does not have a dedicated pathway, and references the technical considerations set up by the US FDA and the local Therapeutic Goods (Medical Devices) Regulations 2002 [133]. 

In addition to the regulatory difficulties, use of 3D-printed mesh has ethical ramifications for service provision and duty of care. To produce 3D-printed mesh, a template is developed based on patient medical imaging data, and the product is created inside a designated 3D printer, using a pre-specified compound [128, 134]. Each of these step may require a proprietary substance, product or service, and involvement of multidisciplinary personnels. Sharing of patient medical data may be required, and the potential for data breach may have serious consequences. These aspects may not necessarily be fully apparent to clinicians, yet they are expected to provide full disclosure to patients and be ethical gatekeepers. Recent medical device litigation, such as the US$1 billion mesh settlement by Becton Dickinson & Co in 2024 [135], have illustrated the need for transparency in medical products and clinical services to which biomedical companies have a vested financial interest. Although clinicians may have the best intentions for patients, use of incomplete or incorrect information may inadvertently lead to harm. Likewise, clinicians should also not be the sole provision of 3D-printing services, as the potential for misconduct and harm to patients to occur in a clinical setting without oversight is high, as illustrated by Foster’s case studies [125]. 

The safest option, is perhaps a middle ground, where 3D-printing services are provided onsite at the healthcare facility via service agreement with a certified manufacturer. Service agreements could be established to provide a comprehensive 3D printing service package, that incorporates provision of printers, materials and trained technicians. This could be an option in minimising the legal issues of ownership of proprietary knowledge and liability of healthcare facilities during manufacturing, quality control and sterilisation of 3D-printed meshes. Responsibility is distributed to key individuals, namely physicians for selection of products; radiologists and technicians for medical imaging scanning; technicians for segmentation of imaging for 3D printing; engineers for product designing and 3D printing; and physicians and sterilisation units for the final preparation, sterilisation and usage of product [128, 129]. Such business models are not new to the healthcare system, and much could be learned by examining the precedent set by the local blood transfusion services, such as the Northern Ireland Blood Transfusion Service and the Australian Red Cross Life Blood^®^. Both services manufacture blood products from patient blood donations under highly regulated yet transparent systems, and ensure all products are accountable, traceable and reportable in the event of adverse reactions.

Patient safety should be foremost when dealing with emerging technology and implantable devices. All 3D-printed hernia mesh should be produced to a high quality that meets national and international standards. An oversight committee, run independently by the national regulatory authority, could provide service accreditation and maintain a robust pre- and post-market surveillance of safety and efficacy. Sharing of patient information, such as DICOM files, should adhere to local privacy policies and data protection requirements, such as the General Data Protection Regulation (GDPR) for nations in the European Union. An encrypted collaborative platform on a local area network with time-limited and personnel-limited access may be required to ensure no unnecessary retention of patient information during the manufacturing process. A designated data compliance team could regulate access and monitor for potential data breach. 3D printing should also take into consideration of waste production, toxicity of waste and environmental impact of polymers and microplastics. Waste should be disposed of in a safe and regulated manner, as dictated by local legislations and international laws.

### Cost-effectiveness of 3D-printed mesh

The true cost of 3D-printed hernia mesh is unknown at this stage, and much of it is considered proprietary knowledge by companies who are attempting to establish themselves within the 3D-printed patient-specific implant market. The first FDA 510(k) clearance for a 3D-printed surgical mesh was only granted in May 2024, awarded to the US-based PrintBio, Inc.’s 3DMatrix™ product (K232602) [136]. Made from polydioxanone monofilament, it has a macroporous non-woven fully-absorbable architecture that has been theorised to reduce complications and healthcare costs in the long term [73]. More information is expected to come to light as products and services of respective companies are approved by the FDA, or equivalent authority.

While functionally not entirely the same, some insights can be gained by examining 3D-printed orthopaedic implants, which have been in use for some years now. The global market for 3D-printed orthopaedic implants was valued at US$1.7 billion in 2024, and is projected to increase to $6.6 billion by 2033 [137]. In Belgium, 3D-printed hip implants have been reported to cost around €8419, comparable to traditional custom-made implants cost of €6002 [138]. Use of 3D-printed hip implants in revisions surgery, compared to traditional implants, can lead to an annual saving of €1,265 with a 5% gain in quality-adjusted life years (QALY) [138]. Similarly, 3D-printed polyether ether ketone (PEEK) cranial implants are reported to cost around US$5,600 to US$20,522 [139]. While such PEEK implants are about 20–133% more expensive than traditional titanium or polymethyl methacrylate (PMMA) implants, they can achieve an excellent successful implantation rate of 93.7%, with minimal complications [139]. 

A hypothetical business model would suggest an initial capital investment for setup, including equipment, materials, engineering staff, and an ongoing cost for maintenance and consumables. Such a model would likely not be significantly different from implementation of robotic surgical platforms [140]. Once in-house systems are operational, repeatable workflows could reduce per-unit cost over time [141]. The raw materials or consumables required for 3D-printed implant product have been reported to be relatively low [142]. If the upfront costs can be offset by the long-term reductions in complications and reoperations, a viable business model may be possible. In the US, hernia recurrence and associated complications currently account for 20% of annual health expenditure spent on managing incisional hernias in both inpatient and outpatient settings [143]. 

Health economic assessments, such as cost-benefit analysis and cost-effectiveness analysis will need to be performed as more information emerges, particularly product details, clinical performance, and patient outcomes. Incorporating long-term cost-offsets into cost-effectiveness analyses could pave the way for organisational reimbursement, and overcome cost-related adoption barriers faced by healthcare facilities [144]. 

### Future directions

3D-printed hernia meshes promises to be an exciting development in tailored hernia care. The potential to use custom-made hernia mesh tailored to individual circumstances, both anatomical shape and tissue environment, could provide an alterative to current therapeutic options. Smart meshes with inbuilt antimicrobial resistance could be the next-generation implants that address the various implantation problems encountered by current clinicians and may improve long-term patient outcomes. The exact parameters of 3D-printed meshes will need to be fine-tuned, and a partnership between surgeons and biomedical engineers at institutions with a strong biomedical engineering program is likely a good foundation to begin with. Early involvement of regulatory bodies and biomedical companies with an interest in developing and providing 3D-printing services at point of care could address the many issues identified in this review.

Likewise, safety and general in-vivo behaviour of 3D-printed meshes should be characterised preclinically, using standardised in-vivo animal models that mimic the human body and facilitate objective comparison, such as the Mesh Integration Index. Long-term outcomes of 3D-printed meshes should be monitored via a clinical quality registry (CQR), as the typical time frame for mesh complications and failure is in the order of 5 years at a minimum [9]. A registry randomised control trial (RRCT), using data from a well-maintained CQR, is an emerging and acceptable alternative to traditional randomised control trials (RCT) in assessing real-world implementation of interventions, particularly over a long period of time that is often not feasible for RCTs due to running costs [145]. 

Hernias, as a disease entity, is complex, and a universal solution for all scenarios is likely not possible [17]. In the current age of evidence-based medicine, data-driven decision-making is becoming increasingly important in delivering individualised care to patients [146]. There is increasing evidence that hernia care is entering a new phase focused on individualised care and clinicians should strive for data-driven bespoke management.

## Supplementary Information

Below is the link to the electronic supplementary material.

Supplementary Material 1

Supplementary Material 2

## Declarations

### Human and animals rights

This research paper reviewed existing literature, and did not involve any human participants or animals.
