# Supplementary material for: To infinity and beyond: the promise of data-driven 3D printing of hernia mesh – a primer for surgeons
Source: Hernia. 2025 Sep 1;29(1):270. doi: 10.1007/s10029-025-03434-4 (PMC12401771; doi:10.1007/s10029-025-03434-4)
Supplement: Supplementary file 1 — Supplementary Material 1 [file 10029_2025_3434_MOESM1_ESM.pdf]

Article: CaleroCastro 2019

SYRCLE

Entry	Judgement	Support for Judgement
1. Sequence generation (selection bias)	High	'… randomly divided into four groups', randomisation method not described.
2. Baseline characteristics (selection bias)	Low	'Forty female Wistar rats (236-281) were randomly divided into four groups …'<br>'The mean basal weight of 40 rats was 251.40+/-1.63g, and no death occurred during surgery or in the postoperative period …'
3. Allocation concealment (selection bias)	Unclear	Not stated
4. Random housing (performance bias)	Unclear	Unclear whether location would have influence on healing ability and outcome
5. Blinding (performance bias)	Unclear	Not stated
6. Random outcome assessment (detection bias)	Unclear	Not stated
7. Blinding (detection bias)	Unclear	Histological results are unlikely to be influenced by lack of blinding, however paper did not state whether pathologist was blinded or not.
8. Incomplete outcome data (attrition bias)	Low	'In all the rats, Type 3 adhesions …'<br>'All the biopsies that were extracted …'
9. Selective outcome reporting (reporting bias)	Low	'…degree of adhesion after placement of the mesh at the subcutaneous level was measured…'<br>'…each [histological] sample was scored as mild, moderate or severe…'
10. Other sources of bias	Unclear	Some possible variations not stated, such as operator variability

Article: Deveci 2024

SYRCLE

Entry	Judgement	Support for Judgement
1. Sequence generation (selection bias)	High	'…animals were divided into four experimental groups randomly…', randomisation method not specified
2. Baseline characteristics (selection bias)	Low	'Body weight, water and feed consumption were similar between groups'
3. Allocation concealment (selection bias)	Unclear	Not stated
4. Random housing (performance bias)	Unclear	Unclear whether housing location would influence outcome
5. Blinding (performance bias)	Unclear	Not stated
6. Random outcome assessment (detection bias)	Unclear	'…all groups were divided into two subgroups…based on the scarification dates of 14th or 28th day after the hernia repair.'
7. Blinding (detection bias)	Unclear	Unclear what affect it will have. Paper does not explicitly states whether assessors were blinded and animals were randomly chosen
8. Incomplete outcome data (attrition bias)	Low	All animals represented in Table 6 and 7. No reports of loss of animals.
9. Selective outcome reporting (reporting bias)	Low	All outcomes were reported and analysed
10. Other sources of bias	High	No other concerns

Article: Erwin 2023

SYRCLE

Entry	Judgement	Support for Judgement
1. Sequence generation (selection bias)	High	No attempt or description of randomisation.
2. Baseline characteristics (selection bias)	Low	'…utilized 10 New Zealand White rabbits aged 6-9 months weighing 1-2kg.'
3. Allocation concealment (selection bias)	High	No attempt or description of randomisation.
4. Random housing (performance bias)	Unclear	Unclear if cage placement will affect individual animal healing capabilities post hernia formation and post-implant insertion.
5. Blinding (performance bias)	Unclear	Unclear if precautions were taken. No descriptions have been provided.
6. Random outcome assessment (detection bias)	Unclear	Not stated in text.
7. Blinding (detection bias)	Low	'..post-surgery implant was performed in individual cages and each rabbit was given several drugs two times a day for 5 days which included antibiotic and anti-inflammatory PO..'
8. Incomplete outcome data (attrition bias)	Unclear	No mentioning of whether all animals were included in the final analysis, or whether any animals had any issues
9. Selective outcome reporting (reporting bias)	Low	Assessment and results match up
10. Other sources of bias	High	'…each rabbit was given several drugs tow times a day for 5 days which included antibiotic and anti-inflammatory PO …' Statement does not specify whether same quantities were given or not.

Article: Hu 2021

SYRCLE

Entry	Judgement	Support for Judgement
1. Sequence generation (selection bias)	High	No mentioning of randomisation process.
2. Baseline characteristics (selection bias)	Unclear	'… male Sprague-Dawley rats with bodyweight of 200g … were provided …' No other mentioning of animal species characteristics.
3. Allocation concealment (selection bias)	Unclear	Not stated in text.
4. Random housing (performance bias)	Unclear	Unclear whether this would have an effect on animal healing and general well being
5. Blinding (performance bias)	Unclear	Not stated in text.
6. Random outcome assessment (detection bias)	Unclear	Not stated
7. Blinding (detection bias)	Low	Unlikely to have influenced outcome
8. Incomplete outcome data (attrition bias)	Unclear	It is implied by the results section that all animals were included in the analysis, however this was not definitively stated by the authors.
9. Selective outcome reporting (reporting bias)	Low	Results and methods match up.
10. Other sources of bias	Unclear	The authors did not state whether or not such replacement occurred or not.

Article: Hu 2022

SYRCLE

Entry	Judgement	Support for Judgement
1. Sequence generation (selection bias)	High	No randomisation process was described by the authors.
2. Baseline characteristics (selection bias)	Unclear	'…male Sprague-Dawley rates of 180-200 g…were used.'
3. Allocation concealment (selection bias)	Unclear	Not stated by the authors in text
4. Random housing (performance bias)	Unclear	Unclear whether healing capabilities could be influenced by cage placement
5. Blinding (performance bias)	Unclear	Not stated by the authors in text
6. Random outcome assessment (detection bias)	Unclear	No stated by the authors
7. Blinding (detection bias)	Low	Likely not influenced by randomisation, as all animals underwent the same set of tests
8. Incomplete outcome data (attrition bias)	Unclear	Results implied all animals were included, but authors did not state whether this occurred or not.
9. Selective outcome reporting (reporting bias)	Low	Expected outcomes were included
10. Other sources of bias	Unclear	Authors did not state whether or not they had drop outs.

Article: Hu 2024

SYRCLE

Entry	Judgement	Support for Judgement
1. Sequence generation (selection bias)	High	Randomisation process not discussed by authors.
2. Baseline characteristics (selection bias)	Unclear	'180-200g of male Sprague-Dawley rats were acquired…'
3. Allocation concealment (selection bias)	Unclear	Not stated by authors within text.
4. Random housing (performance bias)	Unclear	Not stated by authors within text.
5. Blinding (performance bias)	Unclear	Not stated by authors within text
6. Random outcome assessment (detection bias)	Unclear	Not stated by authors whether a random generated sequenced was used or not
7. Blinding (detection bias)	Low	Outcome assessments were the same for all animals, and assessment scores unlikely to be influenced by non-blinding.
8. Incomplete outcome data (attrition bias)	Unclear	Results imply inclusion of all animals, but not clearly stated by authors
9. Selective outcome reporting (reporting bias)	Low	Expected outcomes all reported
10. Other sources of bias	Unclear	Not clearly stated authors whether any replacements occurred or not.

Article: Qamar 2019

SYRCLE

Entry	Judgement	Support for Judgement
1. Sequence generation (selection bias)	High	Randomisation component not described by authors
2. Baseline characteristics (selection bias)	Unclear	Implied by methods that baseline were the safe, though not explicitly stated by the authors
3. Allocation concealment (selection bias)	Unclear	Not stated in the text
4. Random housing (performance bias)	Unclear	Unclear if animal healing abilities could be influenced by variations in housing
5. Blinding (performance bias)	Unclear	Not stated in the text
6. Random outcome assessment (detection bias)	Unclear	Not stated in the text
7. Blinding (detection bias)	Low	Implant performance likely not influenced by lack of blinding
8. Incomplete outcome data (attrition bias)	Low	Number of implants extracted and subsequent results implies all animals were used
9. Selective outcome reporting (reporting bias)	Low	All expected outcomes described
10. Other sources of bias	Unclear	Not stated by authors whether any drops out occurred or not

Article: Russo Serafini 2023

SYRCLE

Entry	Judgement	Support for Judgement
1. Sequence generation (selection bias)	High	Randomisation process not described
2. Baseline characteristics (selection bias)	Unclear	Implied to be the same, though not explicitly stated by the authors
3. Allocation concealment (selection bias)	Unclear	Not stated in text
4. Random housing (performance bias)	Unclear	Unclear whether animal healing and recovery could be affected by housing location or not
5. Blinding (performance bias)	Unclear	Not stated in text
6. Random outcome assessment (detection bias)	Unclear	Not stated in text
7. Blinding (detection bias)	Low	Lack of blinding likely did not affect assessments
8. Incomplete outcome data (attrition bias)	Unclear	Implied by authors to have included all animals, though not explicitly stated
9. Selective outcome reporting (reporting bias)	Low	Planned assessments all reported
10. Other sources of bias	Unclear	Not stated in text whether replacement animals occurred or not

Article: Shin 2021

SYRCLE

Entry	Judgement	Support for Judgement
1. Sequence generation (selection bias)	High	'…rats were randomly divided into two groups …' Randomisation process not described.
2. Baseline characteristics (selection bias)	Unclear	Implied in text, but not explicitly stated by authors
3. Allocation concealment (selection bias)	Unclear	Not stated in text
4. Random housing (performance bias)	Unclear	Unclear whether healing abilities would be influenced by housing randomisation
5. Blinding (performance bias)	Unclear	Not stated in text
6. Random outcome assessment (detection bias)	Unclear	Not stated in text
7. Blinding (detection bias)	Low	Assessments equally applied to all animals
8. Incomplete outcome data (attrition bias)	Unclear	Text implies all animals were included, but not explicitly stated by authors.
9. Selective outcome reporting (reporting bias)	Low	Expected outcomes all measured and reported
10. Other sources of bias	Unclear	No mentioning of whether drop outs occurred or not

Article: Song 2023

SYRCLE

Entry	Judgement	Support for Judgement
1. Sequence generation (selection bias)	High	Randomisation process not described
2. Baseline characteristics (selection bias)	Unclear	Implied by text to be balanced, but not explicitly stated
3. Allocation concealment (selection bias)	Unclear	Not stated in text
4. Random housing (performance bias)	Unclear	Not stated in text
5. Blinding (performance bias)	Unclear	Not stated in text
6. Random outcome assessment (detection bias)	Unclear	Not stated in text
7. Blinding (detection bias)	Low	Assessment were the same in all animals, and outcome unlikely to be affect by lack of blinding
8. Incomplete outcome data (attrition bias)	Unclear	Implied by results to have included all animals, but not explicitly stated by authors
9. Selective outcome reporting (reporting bias)	Low	Expected outcomes all reported.
10. Other sources of bias	Unclear	Not clearly stated by authors whether any drop outs occurred

Article: Shea 2020

ROBINS-I

Entry	Judgement	Support for Judgement
1. Bias due to confounding	Critical	No controls present in study
2. Bias in classification of interventions	Critical	No attempt to screen or classify patients. Retrospective analysis only pools together patients who used a 3D-printed model.
3. Bias in selection of participants into the study	Low	Use of 3D-printed model occurs before start of follow up. Follow up coincides with usage.
4. Bias due to deviations form intended interventions	Low	Study undertaken in experimental context, with insufficient information to determine if deviate from recruitment. Appropriate analysis used for context.
5. Bias due to missing data	Serious	Not all possible cases were included due to lack of follow up or different materials.
6. Bias in measurement of the outcome	Moderate	Retrospective nature results in assessors being aware of intervention received and follow up outcomes
7. Bias in selection of the reported result	Low	Results reported in accordance to methods and analysis plan

Article: Smietanski 2023

ROBINS-I

Entry	Judgement	Support for Judgement
1. Bias due to confounding	Critical	No controls present in study
2. Bias in classification of interventions	Low	Intervention does not rely on events after start of follow up, with information recorded at start, without classification error.
3. Bias in selection of participants into the study	Low	Intervention does not rely on events or characteristics after start of follow up. Follow up coincides with intervention.
4. Bias due to deviations form intended interventions	Low	Study undertaken in experimental context with appropriate non-analysis.
5. Bias due to missing data	Low	All data present
6. Bias in measurement of the outcome	Serious	Outcome assessors aware of intervention and assessment could be influenced by knowledge
7. Bias in selection of the reported result	Low	Results reported according to analysis plan

Article: Ballard 2017

OHAT Risk of Bias

Entry	Judgement	Support for Judgement
1. Selection bias	Probably high	Insufficient information of concealment
2. Performance bias	Probably high	Insufficient information of blinding
3. Detection bias	Probably low	Materials likely contained the necessary antimicrobial properties.
4. Attrition / exclusion bias	Definitely low	All sample results reported
5. Selective reporting bias	Definitely low	All samples reported
6. Other bias	Probably high	A control plate without any mesh was not used. The plates could have been contaminated.

Article: Ballard 2018

OHAT Risk of Bias

Entry	Judgement	Support for Judgement
1. Selection bias	Probably high	No description of attempting to conceal samples
2. Performance bias	Probably high	Not reported
3. Detection bias	Definitively low	Same conditions applied to all samples
4. Attrition / exclusion bias	Definitively low	All samples reported, no exclusion of data
5. Selective reporting bias	Definitively low	All samples reported
6. Other bias	Definitively low	All reasonable controls used and accountable for

Article: CaleroCastro 2019

OHAT Risk of Bias

Entry	Judgement	Support for Judgement
1. Selection bias	Definitively high	No randomisation described
2. Performance bias	Probably high	No information about blinding
3. Detection bias	Definitively low	All exposures same across samples
4. Attrition / exclusion bias	Definitively low	All data reported
5. Selective reporting bias	Definitively low	Intended outcomes all reported
6. Other bias	Probably high	Unclear why Mann-Whitney U was used, instead of t-test. A bare culture plate with nutrient medium only was not used as a control.

Article: Deveci 2024

OHAT Risk of Bias

Entry	Judgement	Support for Judgement
1. Selection bias	Probably high	No description of concealment
2. Performance bias	Probably high	No information
3. Detection bias	Definitively low	Outcome assessments same
4. Attrition / exclusion bias	Probably low	All outcomes appear to have been reported
5. Selective reporting bias	Probably low	Outcomes appear to have been reported
6. Other bias	Probably low	Additional plates could be used to minimise chance of random error

Article: Hu 2021

OHAT Risk of Bias

Entry	Judgement	Support for Judgement
1. Selection bias	Probably high	Not stated in text
2. Performance bias	Probably high	Not stated in text
3. Detection bias	Probably low	Exposure conditions similar across groups
4. Attrition / exclusion bias	Probably low	Text implies all no attrition of samples
5. Selective reporting bias	Definitively low	All outcomes reported
6. Other bias	Probably low	Appears to have accounted of most variables

Article: Hu 2022

OHAT Risk of Bias

Entry	Judgement	Support for Judgement
1. Selection bias	Probably high	Not stated in text
2. Performance bias	Probably high	Not stated in text
3. Detection bias	Probably low	Exposure same for assessments
4. Attrition / exclusion bias	Probably low	No mention of attrition of data in text
5. Selective reporting bias	Probably low	Outcomes appear to have been reported
6. Other bias	Probably low	Controls appear to be present

Article: Hu 2024

OHAT Risk of Bias

Entry	Judgement	Support for Judgement
1. Selection bias	Probably high	Not stated in text
2. Performance bias	Probably high	Not stated in text
3. Detection bias	Probably low	Appears to be same across all samples
4. Attrition / exclusion bias	Probably low	Not stated, but results implies all samples were analysed and included
5. Selective reporting bias	Definitively low	All outcomes reported
6. Other bias	Probably low	Variables accounted for

Article: Olmos-Juste 2022

OHAT Risk of Bias

Entry	Judgement	Support for Judgement
1. Selection bias	Probably high	No mentioning of concealment
2. Performance bias	Probably high	Not stated in text
3. Detection bias	Probably low	Outcome assessments same across samples
4. Attrition / exclusion bias	Probably low	Results imply all data was included, though not explicitly stated by the authors
5. Selective reporting bias	Definitively low	All measured outcomes reported
6. Other bias	Probably low	Appropriate controls and sample variation

Article: Ramos 2023

OHAT Risk of Bias

Entry	Judgement	Support for Judgement
1. Selection bias	Probably high	Not stated in text
2. Performance bias	Probably high	Not stated in text
3. Detection bias	Probably low	Outcome assessment equal
4. Attrition / exclusion bias	Definitively low	All data included
5. Selective reporting bias	Definitively low	All outcomes included
6. Other bias	Probably high	Control samples not used

Article: Shin 2021

OHAT Risk of Bias

Entry	Judgement	Support for Judgement
1. Selection bias	Probably high	No mentioning of randomisation
2. Performance bias	Probably high	Not stated in text
3. Detection bias	Probably low	Outcome assessment adequate
4. Attrition / exclusion bias	Probably low	Data appears to suggest adequate attrition
5. Selective reporting bias	Probably low	Outcomes reported
6. Other bias	Probably high	Controls not used

Article: Song 2023

OHAT Risk of Bias

Entry	Judgement	Support for Judgement
1. Selection bias	Probably high	No stated in text whether any concealment occurred or not
2. Performance bias	Probably high	Not stated in text
3. Detection bias	Probably low	Outcome assessments equal in all
4. Attrition / exclusion bias	Probably high	No stated in text
5. Selective reporting bias	Probably low	All outcomes reported
6. Other bias	Probably low	Control sample not used

Article: Wang 2024

OHAT Risk of Bias

Entry	Judgement	Support for Judgement
1. Selection bias	Probably high	No mentioning of concealment
2. Performance bias	Probably high	Not stated in text
3. Detection bias	Probably low	Assessments were adequate
4. Attrition / exclusion bias	Probably low	All data appears to be present, though not clearly stated by authors
5. Selective reporting bias	Probably low	All outcomes reported
6. Other bias	Probably high	Limited controls

Article: Yadav 2025

OHAT Risk of Bias

Entry	Judgement	Support for Judgement
1. Selection bias	Probably high	Not stated in the text
2. Performance bias	Probably high	No stated in the text
3. Detection bias	Probably low	Outcomes assessed equally
4. Attrition / exclusion bias	Probably low	Does not appear to have been any exclusion
5. Selective reporting bias	Probably low	All measured outcomes reported
6. Other bias	Probably high	No control samples

Article: Yang 2020

OHAT Risk of Bias

Entry	Judgement	Support for Judgement
1. Selection bias	Probably high	Not stated
2. Performance bias	Probably high	Not stated
3. Detection bias	Probably low	Appears to have same outcome assessment
4. Attrition / exclusion bias	Probably low	No reporting of attrition
5. Selective reporting bias	Probably low	All measured outcomes were reported
6. Other bias	Probably low	Appropriate controls
